# Biophysics and Structure-Function Relationships of LRRC8-Formed Volume-Regulated Anion Channels

**DOI:** 10.1016/j.bpj.2019.02.014

**Published:** 2019-02-26

**Authors:** Benjamin König, Tobias Stauber

**Affiliations:** 1 Freie Universität Berlin, Institute of Chemistry and Biochemistry, Berlin, Germany

## Abstract

Volume-regulated anion channels (VRACs) are key players in regulatory volume decrease of vertebrate cells by mediating the extrusion of chloride and organic osmolytes. They play additional roles in various physiological processes beyond their role in osmotic volume regulation. VRACs are formed by heteromers of LRRC8 proteins; LRRC8A (also called SWELL1) is an essential subunit that combines with any of its paralogs, LRRC8B–E, to form hexameric VRAC complexes. The subunit composition of VRACs determines electrophysiological characteristics of their anion transport such as single-channel conductance, outward rectification, and depolarization-dependent inactivation kinetics. In addition, differently composed VRACs conduct diverse substrates, such as LRRC8D enhancing VRAC permeability to organic substances like taurine or cisplatin. Here, after a recapitulation of the biophysical properties of VRAC-mediated ion and osmolyte transport, we summarize the insights gathered since the molecular identification of VRACs. We describe the recently solved structures of LRRC8 complexes and discuss them in terms of their structure-function relationships. These studies open up many potential avenues for future research.

## Main Text

Cells possess the ability to regulate their volume upon osmotic perturbations and can adjust it during physiological processes including proliferation, cell migration, and apoptosis (1). Important players for cellular volume decrease in vertebrate cells are volume-regulated anion channels (VRACs) (2). These channels open in response to osmotic swelling of the cell and facilitate regulatory volume decrease by releasing chloride ions and various organic osmolytes. Currents mediated by VRACs have been observed in all studied vertebrate cells. Only 5 years ago, with the identification of LRRC8 proteins as essential subunits of VRACs, was their molecular identity uncovered (3, 4).

Numerous studies using pharmacological inhibition with moderate selectivity of VRACs before their molecular identification suggested an involvement of VRACs in many physiological and pathological processes, including cell volume regulation, cell division and migration, apoptosis, cancer drug resistance, and inflammation (5, 6, 7, 8, 9, 10, 11). The discovery of LRRC8 heteromers as essential subunits has laid the groundwork for investigations into the physiological roles by molecular biological approaches. Recent studies have supported proposed roles in apoptosis, signaling from astrocytes, and insulin release (12, 13, 14, 15, 16, 17, 18). The severe phenotypes of mice and zebrafish deficient in the physiologically essential VRAC subunit LRRC8A demonstrate its physiological importance (19, 20). Further studies on mouse models with the tissue-specific deletion of LRRC8A and on the spontaneous mutant *ébouriffé* with a truncation of LRRC8A impairing VRAC function suggest that LRRC8A has roles in a variety of processes, including fertility and insulin signaling (11, 21, 22, 23).

### Investigation of VRAC currents before the molecular identification of VRACs

In the few years after their molecular identification (3, 4), tremendous insight into the biophysics of VRACs was obtained using molecular biological tools in mammalian cells and *Xenopus* oocytes (24, 25, 26, 27, 28, 29). VRACs were reconstituted from purified LRRC8 complexes in droplet lipid bilayers (24), and the structures of LRRC8 complexes have recently been resolved (30, 31, 32, 33). The physiological functions of VRACs and the biophysical properties of their currents were, however, already studied extensively decades before the identification of VRAC subunits. After the first observations of swelling-induced anion permeability (34), electrophysiological measurements in different vertebrate cell types revealed common basic properties of VRAC-mediated chloride currents as reviewed previously (5, 8, 35, 36, 37). The observed cell-type-specific differences can now be explained by the potential consequences of differential LRRC8 subunit composition.

VRACs begin to activate within seconds after cell swelling is induced, and it can take up to several minutes for VRAC currents to reach their maximum. In addition, VRACs can be activated by various cues under isotonic conditions. Despite ample investigation, the activation mechanism of VRACs has remained unclear (1, 5, 11, 37). Volume sensing may involve the membrane cytoskeleton or membrane stiffness and composition (37, 38). Importantly, the intracellular ionic strength plays a central role in the activation of VRACs (comprehensively reviewed in (39)). After the identification of its regulatory role (40), further studies reported that the reduction in intracellular ionic strength concomitant to osmotic cell swelling, rather than cell volume changes per se, directly activates VRACs (41). This notion was corroborated by the reported activation of reconstituted VRACs by low ionic strength (24) (see below). However, cell volume alterations by fluid injection or withdrawal without changes in ionic strength activated or inactivated VRACs in various studies (39); ionic strength may, through an unknown mechanism, control the volume set point for VRAC activation (42, 43). Various other signaling pathways were reported to facilitate VRAC activation, often under isotonic conditions. These include G-protein-coupled receptors, purinergic signaling, reactive oxygen species, calcium signaling, and phosphorylation cascades (8, 37, 38, 44).

**Figure 1:**
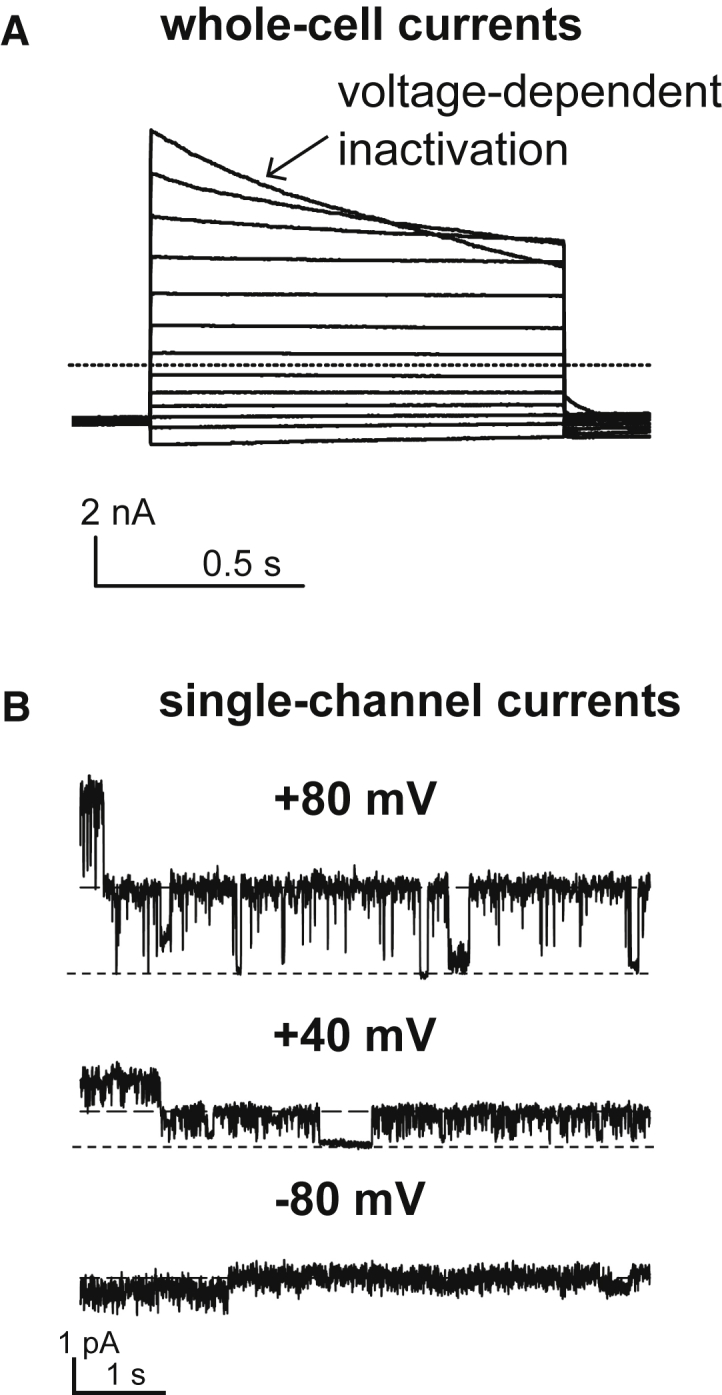
VRAC-mediated currents. (*A*) Current traces (*dotted line* indicates zero current) of hypotonicity-activated VRAC currents by native VRAC in human embryo kidney cells are shown, measured in 20-mV steps between −120 and 120 mV, showing outward rectification and voltage-dependent inactivation at positive potentials. From (4). (*B*) Single-channel currents at indicated voltages from *Xenopus* oocytes expressing fluorescently tagged LRRC8A and LRRC8E are shown. From (28).

Single-channel conductance of VRACs was measured as 50–80 and 10–20 pS at positive and negative membrane potentials, respectively (45, 46, 47, 48). Hence, whole-cell currents display moderate outward rectification, for which VRACs are also named volume-sensitive outwardly rectifying anion channels. At positive potentials, they display variable time-dependent inactivation (Fig. 1 *A*) because individual channels successively close at these voltages (49). Nonstationary noise analysis of such inactivating VRAC currents revealed larger unitary conductance than the earlier stationary noise analysis of brief current recordings during VRAC activation, with 15–20 pS vs. ∼1 pS at 0 mV (48). This discrepancy may be caused by a stepwise, rather than gradual, increase of the open probability during VRAC activation by distinct VRAC populations because of their differential configuration or local environment (39).

The permeability sequence corresponds to Eisenman’s sequence I, which has a selectivity sequence of I^−^ > Br^−^ > Cl^−^ > F^−^ for an anion binding site of weak field strength (37). In addition to these inorganic ions, VRACs were reported to conduct larger organic osmolytes like taurine, *myo*-inositol, glutamate, and even ATP (50, 51). Therefore, they were alternatively named volume-sensitive organic osmolyte/anion channels. The debate as to whether volume-sensitive organic osmolyte/anion channels and VRACs are indeed the same entities (52, 53) may retrospectively be solved by the notion that VRACs with different LRRC8 subunit compositions display diverse substrate preferences (discussed in more detail below). Permeability measurements with different reporters suggested a pore size of 11–17 Å (54, 55).

### VRACs are formed by LRRC8 heteromers with variable biophysical properties

#### LRRC8 heteromers as pore-forming components of VRACs

Five years ago, LRRC8A was identified as an essential component of VRACs by two independent genome-wide small interfering RNA screens (3, 4). Heteromerization of LRRC8A with at least one other member of the LRRC8 protein family (LRRC8B-E) was required for detectable VRAC activity (4). The five LRRC8 paralogs consist of ∼800 amino acids. Their amino-terminal half contains four TMDs, whereas their carboxy-terminal half comprises a leucine-rich repeat domain (LRRD) of up to 17 leucine-rich repeats (LRRs), hence the family name of LRR-containing protein family 8 (56, 57). The predicted topology with cytosolic amino- and carboxy-termini (58) was confirmed by domain accessibility to antibodies and protease and by the confirmation of N-linked glycosylation in the extracellular loop (EL) between transmembrane helices (TMHs) TMH2 and TMH3 (3, 4, 5). The LRRC8 family shares this topology and also analogous cysteines in the ELs with calcium homeostasis modulators, connexins, and pannexins, as well as their invertebrate homologs innexins. In addition, weak sequence homology between pannexins and LRRC8 proteins (58) and the overlapping pharmacological profile, with carbenoxolone and 4-[(2-butyl-6,7-dichloro-2-cyclopentyl-2,3-dihydro-1-oxo-1*H*-inden-5-yl)oxy]butanoic acid (DCPIB) inhibiting both channel types (60, 61, 62, 63), further corroborates their relationships. Because pannexins, connexins, and calcium homeostasis modulators form hexameric channels (64, 65, 66), LRRC8s were also predicted to form hexamers (58). Heteromerization of LRRC8A with the other LRRC8 members was shown by coimmunoprecipitation and subcellular cotrafficking (4, 24, 59). Results from cross-linking and gel electrophoresis (24) and stepwise bleaching of LRRC8 complexes composed of subunits fused to fluorescent proteins in *Xenopus* oocytes (28) were compatible with hexameric complexes, as has recently been confirmed in cryo electron microscopy (cryo-EM) structures (30, 31, 32, 33) (see below).

Two lines of experiments provided the first evidence for the involvement of LRRC8 proteins in the pore formation of VRACs. Firstly, when LRRC8A was heterologously expressed to restore VRAC currents in knockdown cells, mutation of threonine T44 in the first TMH (TMH1) of LRRC8A altered the permeability ratio for Cl^−^ and I^−^ (3), which was later also shown for the equivalent threonines in LRRC8C, -D, and -E (24). Further evidence arose from the expression of LRRC8A in different combinations with other LRRC8 proteins (4). This was achieved by either heterologous coexpression in cells depleted of all five LRRC8 genes or by depleting different combinations of LRRC8B-E, aside from the combined knockout of all of LRRC8B-E, which yielded no detectable VRAC currents. Depending on the subunit combination, VRAC currents displayed differences in voltage-dependent inactivation kinetics, an expected channel-intrinsic property, placing the LRRC8 proteins close to the pore (4). Using chimeras between LRRC8C and LRRC8E, which in coexpression with LRRC8A showed particularly slow and fast inactivation at inside-positive voltages, respectively, few charged amino acids close to TMH2 in the first extracellular loop (EL1) were found to determine the kinetics and voltage dependence of this inactivation (25). Remarkably, charge reversal of the first of these amino acids (K98 in human LRRC8A and at equivalent positions in LRRC8C and -E) additionally reduced the permeability selectivity of I^−^ > Cl^−^, suggesting an involvement of these residues in the outer pore (25). Similar alterations were observed with (functional homomeric) chimeras of LRRC8A with EL1 partly replaced by that of LRRC8E (27). Recent resolutions of LRRC8 complexes (see below) have confirmed a role of this EL1 region in channel pore formation (30, 31, 32, 33). Introducing a stretch of 25 amino acids from the intracellular loop (IL) between the second and third TMH of LRRC8A into LRRC8C or -E also yielded (functional homomeric) chimeras with altered ion permeability, voltage-dependent inactivation, and rectification, implicating this region in pore function (27).

#### Differential subunit compositions underlie diverse VRAC properties

A further fundamental biophysical property of VRACs determined by their subunit compositions, besides inactivation kinetics, is single-channel conductance. Purification of LRRC8 complexes from culture cells and reconstitution in droplet lipid bilayers enabled the measurement of flickering single-channel currents of VRACs. Their mixture of conductances in the ranges of 40–80 pS at +100 mV and 10–40 pS at −100 mV (24) are in agreement with previous measurements of native VRAC currents (45, 46, 47, 48), which may be underestimated (see above), and with single-cell currents of heterologously expressed LRRC8 heteromers in *Xenopus* oocytes (Fig. 1 *B*) (28). When specific combinations of LRRC8A with another LRRC8 from a cell line depleted of the other three LRRC8 genes were measured, different combinations yielded different single-channel conductances, each a subset of the mixed population from wild-type cells (24). The rectification of single-channel currents was the same for different subunit compositions. However, the study reported subunit-composition-dependent differences in the open probability (24), explaining the stronger rectification of LRRC8A/D whole-cell currents (24, 27). Furthermore, subunit composition-specific changes in of I^−^ > Cl^−^ selectivity were described. Whereas the differences in I^−^ > Cl^−^ selectivity are not as prominent in other studies (4, 28), the differences between subunits in the transport of osmolytes are drastic.

Interestingly, LRRC8A alone gave VRAC currents in the bilayer system with low single-channel conductance (24), although LRRC8A expressed without another LRRC8 yielded no detectable whole-cell currents in this and earlier studies (4, 24). Overexpression of LRRC8A even suppressed endogenous VRAC currents (3, 4, 24), whereas VRAC currents were increased by reducing the relative amount of LRRC8A in LRRC8 mixtures (20). Single-channel currents were measured with constituted LRRC8A in further studies (31, 33). Furthermore, small currents by LRRC8A expressed alone in LRRC8-deficient cells have meanwhile been reported in several independent studies (27, 30, 32). However, these currents were relatively volume insensitive (27) and activated only by large reductions in intracellular ion strength (30). Although LRRC8A homomers do not recapitulate native VRAC properties, the necessity of LRRC8 heteromerization could be overcome by replacing the first EL of LRRC8A with that of LRRC8C or by inserting the IL between the second and third TMH of LRRC8A into another LRRC8. Although partly exhibiting altered permeability, rectification, and depolarization-dependent inactivation, these chimeras displayed normal regulation by osmotic volume changes (27).

The molecular identification of LRRC8 proteins as essential VRAC components enabled the unambiguous demonstration that swelling-induced osmolyte release is indeed mediated by VRACs (3, 4). Since then, VRAC-mediated transport of a wide range of solutes has been confirmed through LRRC8 depletion, including taurine and *myo*-inositol; the amino acids aspartate, glutamate, serine, lysine, and glycine; and ATP (3, 4, 15, 28, 67, 68, 69). Additionally, LRRC8s were found to facilitate at least part of the cellular uptake of the antibiotic blasticidin and the anticancer drug cisplatin, in which LRRC8D plays a major role (13, 59). Whereas coexpression of LRRC8A with any other LRRC8 protein allows for anion transport, combination of LRRC8A with LRRC8D is critical for the transport of larger uncharged (taurine, *myo*-inositol, GABA, glycine) or positively charged (lysine) substances (13, 28, 68). Negatively charged substrates like aspartate required LRRC8A/D, LRRC8A/E, or LRRC8A/C (68, 69). ATP permeated better when LRRC8A was coexpressed with LRRC8E than with LRRC8C (28). Only for LRRC8B has no transport ability beyond inorganic ions been described so far.

Subunit stoichiometries of native VRACs are unknown, but there are many differential subunit combinations possible for a hexamer. Sequential coprecipitation has revealed that LRRC8A can combine with more than one other type of LRRC8 to form one complex (68), and stepwise subunit bleaching has shown variable subunit ratios depending on the relative expression levels (28). The variations in substrate selectivity likely form the basis of differential physiological functions for differentially composed VRACs in various cell types and organs (4, 15, 18, 22, 68, 69), and they may resolve the previous controversies as to whether VRACs and VSOACs are the same entities (52, 53). In addition, the potential variety of VRACs with different LRRC8 combinations may in part explain the obscurity of the regulatory mechanisms. When expressed in *Xenopus* oocytes, VRACs composed of LRRC8A/E were found to be activated by cysteine oxidation, whereas LRRC8A/C and LRRC8A/D heteromers were inactivated (70). Most subunit composition-specific properties relate to pore-intrinsic features of channels such as voltage-dependent inactivation kinetics, single-channel conductance, and substrate selectivity. Together with point mutations altering ion selectivity (3, 24, 25, 26) and the successful reconstitution of VRAC currents by purified LRRC8 complexes in lipid bilayers (24), this demonstrates that LRRC8 complexes are indeed the pore-forming unit of VRACs.

### Insights from solved LRRC8 channel structures

**Figure 2:**
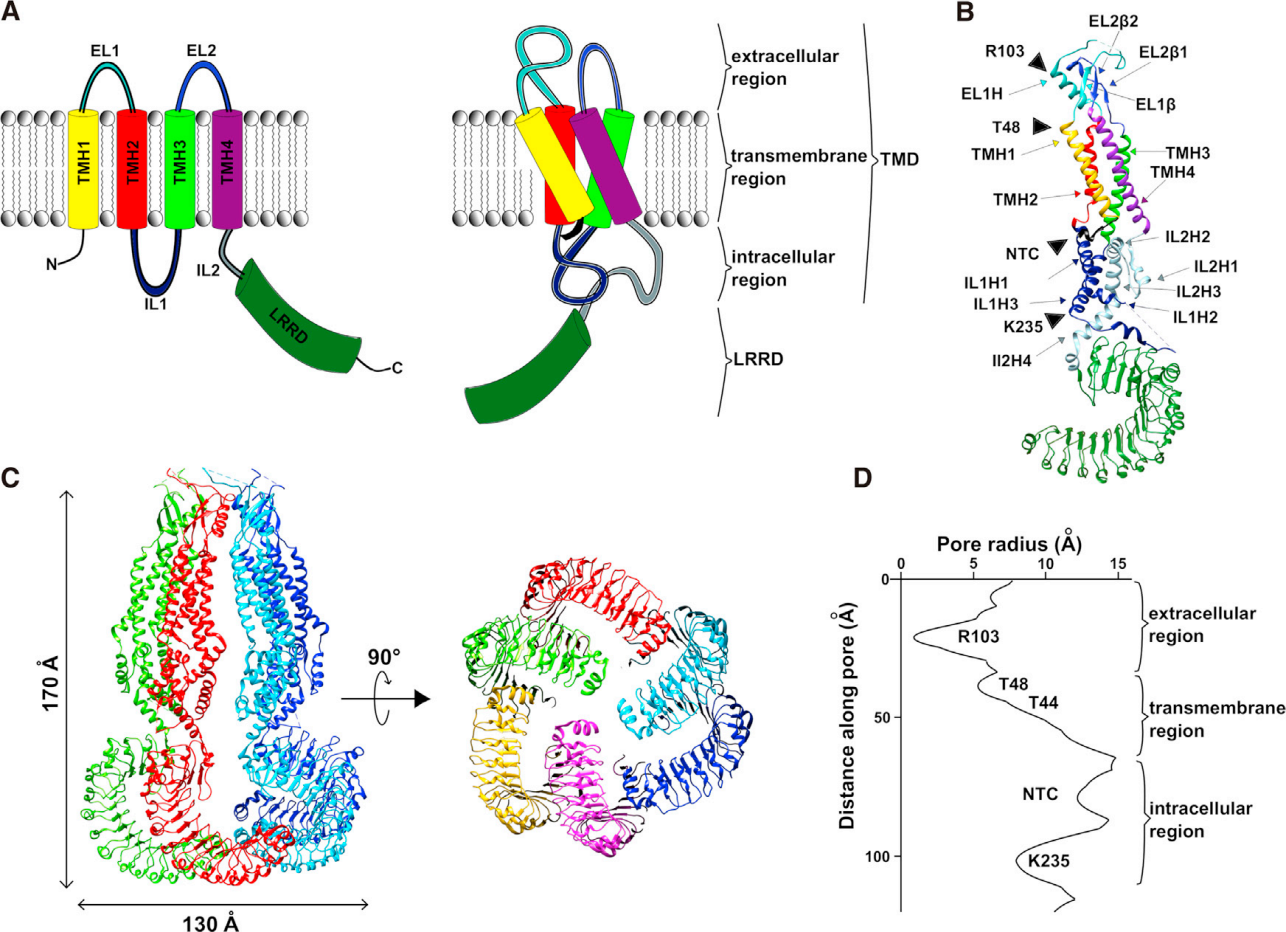
Structure of LRRC8 complexes. (*A*) A schematic representation of the predicted LRRC8 protein topology (58) (*left*) and of the structure of a single LRRC8 subunit in an LRRC8 hexamer (30, 31, 32, 33) (*right*) is shown. (*B*) Ribbon representation of an LRRC8A subunit from a hexamer (Protein Data Bank [PDB]: 6DJB, (32)) is shown. Structural features are illustrated using the same color code as in (*A*). EL1H, TMH1, TMH2, IL1H1, and IL1H3 face the channel pore to the left of the subunit. Only a short carboxy-terminal portion of the NTC is resolved. Unresolved stretches connecting EL1*β* with EL1H and IL1H2 with IL1H3 are depicted as dashed lines. (*C*) Structures of an LRRC8A hexamer (PDB: 5ZSU, (31)) are shown, viewed parallel to the membrane with two subunits in the back not shown for clarity (*left*) and from the intracellular side (*right*), with individual subunits distinguished by different colors. Note the C3 symmetry (trimer of dimers) of the LRRD. (*D*) Pore radius along the symmetry axis is shown with the position of key amino acids depicted. The unresolved NTC may reach into the dilation. To see this figure in color, go online.

Approximately 4 years after the molecular identification of VRACs, four independent studies solved the structure of LRRC8A complexes at ∼4 Å resolution by cryo-EM within a short time (30, 31, 32, 33) (Fig. 2; Table 1). LRRC8A expressed alone was found to mediate small whole-cell currents, albeit not recapitulating all properties of VRACs (27, 30, 32), and reconstituted LRRC8A yielded outwardly rectifying single-channel currents in hypotonicity or reduced ion strength (24, 31, 33). Hence, LRRC8A homomers technically form functional channels. Deneka and colleagues additionally solved the structure of LRRC8A/C heteromers at ∼8 Å resolution (30). All structures confirm that LRRC8 proteins assemble into hexameric complexes, elucidate properties of the pore, and allow for speculation about the role of the cytoplasmic LRRD.

**Table 1:** Comparison of Published VRAC Structures

	Deneka et al. (30)	Kasuya et al. (31)	Kefauver et al. (32)	Kern et al. (33)
Subunit composition	LRRC8A homomer; LRRC8A/C heteromer	LRRC8A homomer	LRRC8A homomer	LRRC8A homomer
Organism	mouse	human	human	mouse
Functionality of LRRC8A homomer	small whole-cell currents with compromised activation by low ionic strength upon expression in LRRC8-deficient cells	single-channel currents in liposomes with reconstituted LRRC8A under asymmetric ion and osmolarity conditions	swelling-induced whole-cell currents upon expression in LRRC8-deficient cells	single-channel currents in excised patch from lipid with reconstituted LRRC8A
Symmetry TMD	C6	C3	C6	C6
Symmetry LRRD	C3	C3 (compact); disordered (relaxed)	C3	disordered
Preparation	digitonin	digitonin	digitonin	nanodisks; digitonin
Different states	no	“compact” versus “relaxed”	no	“constricted” versus “expanded”

#### Domain structure of the LRRC8 subunits

The channel, as well as each subunit, consists of a transmembrane pore domain (TMD) formed by the amino-terminal part and an LRRD shaped by the carboxy-terminal part (30, 31, 32, 33). The TMD contains of an extracellular region, a transmembrane region, and an intracellular region (Fig. 2 *A*). As predicted, the overall architecture is similar to the gap-junction channels innexins and connexins (58, 71, 72).

In a single subunit, the extracellular region is formed by the two ELs (EL1 and EL2) connecting the TMHs. EL1 consists of a *β*-strand (EL1*β*) and an *α*-helix (EL1H). The amino-acid stretch between these, where LRRC8A is N-glycosylated (4), is unresolved in all structures (30, 31, 32, 33). EL2 contains two *β*-strands (EL2*β*1 and EL2*β*2), forming an antiparallel *β*-sheet with EL1*β* (Fig. 2 *B*). Three disulfide bridges stabilize the extracellular region. As predicted, four TMHs (TMH1–TMH4) span the plasma membrane to shape the transmembrane region. TMH1 faces the lumen of the channel, where TMH3 and TMH4 are directed toward the membrane (Fig. 2 *B*). The amino-terminus likely stretches from the cytosolic side into the pore (30, 31, 32, 33). It is predicted to form an amino-terminal coil (NTC), as it does in connexins and innexins (71, 72). The first 14 amino acids at the extreme amino-terminus are, however, unfortunately not resolved in any of the published structures. The intracellular region of the TMD on the cytoplasmic side of the channel is formed by two ILs, IL1 and IL2 (Fig. 2 *B*). IL1, the IL between TMH2 and TMH3, contains three *α*-helices with an unresolved stretch between the second and third helices that harbors several putative phosphorylation sites (58). Remarkably, LRRC8C homomers were rendered functional when this unresolved region was replaced with the equivalent sequence from LRRC8A (27). IL2 connects TMH4 and the LRRD and contains four to five *α*-helices (Fig. 2 *B*).

Because the LRRDs are specific to VRACs compared to connexins and innexins (71, 72), they are of particular interest for their structure-function relationship, especially considering their unusual cytosolic localization (58). The LRRD forms the typical horseshoe structure known for this type of domain, with 16 repeats of an *α*-helix and a *β*-strand, as seen in the x-ray structure of the isolated domain (30). The concave side of the horseshoe faces the lumen of the channel (30, 31, 32, 33).

#### Symmetry of the channel structure

The LRRC8 complexes span 165–180 Å along the pore axis perpendicular to the membrane and have a maximal diameter of 110–130 Å parallel to the membrane in the LRRD (Fig. 2 *C*). They contain six subunits with individual TMDs packed separately without intertwining. There are inconsistencies in the reported symmetries (Table 1). Most structures, irrespective of preparation in digitonin or lipid nanodisks, display sixfold rotational C6 symmetry for the whole TMD, including the extracellular, transmembrane, and intracellular regions with respect to the center of the pore (30, 32, 33). In this likely structure, all interfaces between neighboring subunits are undistinguishable. In contrast, Kasuya and colleagues reported a C3 symmetry with pairs of the subunits having tight interfaces and building a trimer-of-dimer arrangement (31).

The arrangement of the LRRD is equally ambiguous in the solved structures (Table 1). Three studies report C3 symmetry with pairs of LRRDs interacting tightly with each other and more loosely with neighboring subunits (30, 31, 32). The pairs are rotated by roughly 40° toward the next pair, and all are tilted toward the membrane by 30–40° with respect to the pore axis (Fig. 2 *C*). This trimer-of-dimer arrangement would expand the possibilities for differential interaction within heteromers because the position of a subunit within the complex influences its biophysics. Even among these structures, all of which were modeled from digitonin-solubilized VRACs, a subset of particles showed a heterogenous arrangement of the LRRD (30, 31, 32). Interestingly, the structure with proteins in nanodisks showed only disordered or at least very heterogenous LRRDs with respect to each other (33). Together, this suggests high flexibility and various orientations of the LRRDs within a VRAC.

Apart from the symmetry, the overall compactness of the channels differs between structures (Table 1). It was hypothesized that lower salt concentrations during sample preparation underlie the observed looser interaction, reflecting the transition of VRACs from a compact closed conformation to an intermediate open state (31). Besides, a subset of more loosely packed particles presented no C3 symmetry of the LRRD. A subgroup of the complexes in nanodisks was also relaxed, implying there is a conformational change in the channel upon activation (33). This would lead to a generally wider pore with the LRRDs moved away from the pore axis.

#### The channel pore

All four reports (30, 31, 32, 33) agree on the architecture of the pore (Fig. 2 *D*). It is lined with hydrophilic and positively charged amino acids. When viewed from the extracellular side, the first resolved part of the pore, ∼25 Å above the membrane, is formed by a ring of arginines, R103, of EL1H (Fig. 2, *B* and *D*). This is the narrowest constriction, with diameters ranging from 6 to 7.6 Å depending on the structure. This arginine lies in the region previously found to determine the inactivation kinetics (25, 27) and was found to be important for the selectivity of VRACs between anions and blockage by extracellular ATP (25, 27, 30, 32). Kern and colleagues were able to solve the structure of VRAC, together with its inhibitor, DCPIB, and found that R103 is involved here as well (33). The hydrophobic part of DCPIB does not fit through this constriction and instead remains stuck, preventing ion flux through the pore. It will be interesting to see whether mutagenesis of R103 or functional homomeric LRRC8 chimeras lacking this residue (27) are indeed insensitive to DCPIB. Although these chimeras also retain the selectivity of Cl^−^ over Cs^+^ typical for VRACs (27), the measurable Na^+^ permeability of LRRC8A/C heteromers with R103 in LRRC8A (LRRC8C has a leucine at this position) replaced with alanine suggested the involvement of R103 in charge selectivity (30).

Further down, the pore is predominantly formed by TMH1 and by TMH2 in the lower part of the transmembrane region (30, 31, 32, 33) (Fig. 2 *B*). Two threonines of TMH1 line the pore. T44 was previously reported to contribute to ion selectivity (3, 24), but T48 reaches further into the pore (Fig. 2 *D*). An observable dilation can be seen where the NTC probably protrudes into the pore, further narrowing it, as is true for connexins and innexins (71, 72). Indeed, mutations in the amino-termini of contributing LRRC8A/C proteins in heteromers have drastic effects on the functionality, rectification, halide selectivity, and inactivation of the formed VRACs (26). Mutations of the first amino acids, I2, P3, and V4, in either subunit nearly abolished detectable currents. Cd^2+^ block of cyteine mutants E6C and R8C suggest close proximity of these residues within the pore (26), and substituted-cysteine accessibility method analysis showed blockage of the channel with applied thiol-reactive reagents (26, 32). These findings underline the high probability that like in connexins (72), in VRACs, the amino-termini form a funnel, narrowing the channel pore.

Further toward the cytosolic side, the pore is formed by a meshwork of helices of the intracellular region (30, 31, 32, 33). Here, another constriction is formed by K235 of IL1H3 (Fig. 2, *B* and *D*). Because of their likely flexible nature, the influence of the LRRD on the formation of the pore is hard to estimate yet.

#### Heteromerization

Homomers of LRRC8A are not known to play a role in cellular context but rather need other members of the LRRC8 family to form functional channels (4, 24, 28). Deneka and colleagues solved the structure of complexes of LRRC8A and LRRC8C copurified in similar amounts (30). Although these complexes may exhibit a variation of LRRC8A/C stoichiometries with unknown functionality, they contained LRRC8A/C heteromers that gave VRAC currents. The resolution of the solved structure was high enough to confirm that the hexameric assembly also holds true for mixtures of different family members, and their structures follow the same architecture as described for LRRC8A homomers (30).

Mapping conservation scores of all LRRC8 proteins on the structure of LRRC8A homomers reveals that the pore formed by the extracellular region, TMHs, and the intracellular region is highly conserved, highlighting that the overall pore formation is the same for various combinations of LRRC8 proteins (31). Functional diversity thereby arises because of the diversified regions. Besides TMH3 and TMH4 facing the lipid environment, the surface of the extracellular region facing the extracellular space, and the LRRD in general, these comprise the amino-terminus, which seems to be involved in pore formation (26), and the amino acids at the constriction formed by R103 in LRRC8A.

### Future directions

With the molecular identification of LRRC8 proteins as essential subunits (3, 4) and especially now with the general structure of VRACs at hand (30, 31, 32, 33), structure-function studies will certainly elucidate many aspects of the exciting biophysics of VRACs. Urgent questions concern the molecular determinants of substrate specificity brought about by different subunit combinations and the gating mechanisms. Except for the constrictions by the extracellular pore entrance at R103 in LRRC8A and probably the amino-termini, the VRAC pore is relatively wide (30, 31, 32, 33), allowing for the transport of different anions and even larger osmolytes (3, 4, 13, 28, 68, 69). Many mutations are tolerated for Cl^−^ transport, but some amino acids have been identified to play a role in substrate selectivity (3, 24, 25, 26, 30, 32). Future work on the structure-function relationship will reveal interesting details in the functional diversity enabled by the differences in these residues between the subunits. For example, the bulky charged side chain of R103 in LRRC8A at the narrowest constriction is replaced by L105, F143, and L106 in LRRC8C, -D, and -E, respectively. Heteromerization with unclear stoichiometry and subunit arrangements complicates such studies. The requirement of the amino-terminus for VRAC functionality (26) likely hampers the use of concatemers. Instead, chimeric proteins that function as homomers (27) may be useful tools for functional studies. Such constructs may also help to resolve the structures of differentially formed pores that allow permeation of larger osmolytes. Interestingly, many mutations affected both ion selectivity and gating (as seen during the voltage-dependent inactivation) of the channels (25, 26, 27). To understand the coupling of these parameters, structural comparison of closed and fully open pores—ideally with the amino-terminus in a fixed and resolvable conformation—would be desirable.

A further intriguing question concerns the activation mechanism of VRACs. Lowered ionic strength was sufficient to activate reconstituted VRACs in a lipid bilayer context (24). It has been hypothesized that the interaction of charged amino acids in an LRRD with the LRRD of a neighboring subunit is affected by surrounding ions, transmitting the signal of ionic strength in the cytosol to the rest of the channel by steric rearrangements, in agreement with the apparent flexibility of the cytoplasmic LRRD (30, 31, 32, 33). Remarkably, in this context, the fusion of fluorescent proteins to the carboxy-termini in LRRC8 heteromers altered the osmolarity dependence of VRAC activation, rendering them partly active already in isotonicity (28). This, however, also occurred when parts of EL1, which do not directly sense cytosolic ionic strength, were exchanged (27). On the other hand, in a cellular context, there must be more than merely a reduction in intracellular ionic strength to activate VRACs. Further limiting factors are likely involved because the overexpression of functional LRRC8 heteromers did not increase swelling-induced chloride currents above wild-type levels (4), and different cells with similar LRRC8 expression levels showed differences in VRAC activity (73). In addition, VRACs can be activated under isovolumetric conditions, e.g., by cisplatin or sphingosine-1-phosphate (13, 44, 74), likely by mechanisms not involving changes in ionic strength. It will be exciting to see whether post-translational modifications or binding partners of LRRC8 proteins play a role. In this respect, potential interaction partners of the LRRDs or influence of the membrane composition (38) on VRAC activity could furthermore bring about subunit-composition-specific VRAC regulation because the LRRDs and the membrane-facing residues of TMH3 and TMH4 are particularly variable between the paralogs. These future studies will be of high relevance for both the biophysics and the physiology of this fascinating class of ion channels.
